# Timing and documentation of key events in neonatal resuscitation

**DOI:** 10.1007/s00431-018-3160-8

**Published:** 2018-04-30

**Authors:** Adam Charles Heathcote, Jacqueline Jones, Paul Clarke

**Affiliations:** 1 Neonatal Intensive Care Unit, Norfolk and Norwich University Hospitals NHS Foundation Trust, Norwich, NR4 7UY UK; 2 Norwich Medical School, University of East Anglia, Norwich Research Park, Norwich, NR4 7TJ UK

**Keywords:** Neonatology, Resuscitation, Umbilical venous catheter, Medicolegal

## Abstract

Only a minority of babies require extended resuscitation at birth. Resuscitations concerning babies who die or who survive with adverse outcomes are increasingly subject to medicolegal scrutiny. Our aim was to describe real-life timings of key resuscitation events observed in a historical series of newborns who required full resuscitation at birth. Twenty-seven babies born in our centre over a 10-year period had an Apgar score of 0 at 1 min and required full resuscitation. The median (95% confidence interval) postnatal age at achieving key events were commencing cardiac compressions, 2.0 (1.5–4.0) min; endotracheal intubation, 3.8 (2.0–6.0) min; umbilical venous catheterisation 9.0 (7.5–12.0) min; and administration of first adrenaline dose 10.0 (8.0–14.0) min.

*Conclusion*: The wide range of timings presented from real-life cases may prove useful to clinicians involved in medical negligence claims and provide a baseline for quality improvements in resuscitation training.

**Table:** 

**What is Known:** *• Only a minority of babies require extended resuscitation at birth; these cases are often subject to medicolegal interrogation* *• Timings of key resuscitation events are poorly described and documentation of resuscitation events is often lacking yet is open to medicolegal scrutiny*
**What is New:** *• We present a wide range of real-life timings of key resuscitation events during the era of routine newborn life support training* *• These timings may prove useful to clinicians involved in medical negligence claims and provide a baseline for quality improvements in resuscitation training*

## Introduction

The European Resuscitation Council and the American Heart Association publish evidence-based resuscitation algorithms that provide guidance to practitioners involved in newborn resuscitation [13, 14]. However, there is limited guidance on recommended timings by which key resuscitation interventions should be achieved. For example, the Textbook of Neonatal Resuscitation recommends starting positive pressure ventilation within or at 1 min and to administer cardiac compressions for 45–60 s after 30 s of effective positive pressure ventilation if the heart rate remains < 60/bpm [10]. Although suggested timings for the initial basic interventions are sometimes provided in guidelines, timings for the more advanced interventions are not, and furthermore, such timings have not been assessed in the real-life situation. Most babies born in poor condition respond favourably to simple manoeuvres. Fewer than 1% require extended resuscitation involving cardiac compressions, endotracheal intubation, central venous catheterisation and emergency drug administration [6]. Real-life timings for when such key events are achieved in clinical practice are not yet well described.

In 2004–2005, 247 individual claims regarding cerebral palsy/brain injury were reported by the National Health Service (NHS) Litigation Authority of the United Kingdom (UK), accounting for a total value of £363 million (~ €415 million) [5]. By 2016–2017, the number of claims had fallen to 232 yet their total value had increased drastically, to £1.9 billion (~ €2.2 billion) [5]. These high-value claims, many of which concern neonatal brain injury, often feature prolonged resuscitations, the timings within which are subject to intense scrutiny by medicolegal experts and clinical negligence lawyers.

In 2015, the UK’s Secretary of State for Health announced a National Maternity Ambition to reduce the rate of stillbirths, neonatal deaths, maternal deaths and brain injuries occurring during or soon after birth by 20% by 2020, and by 50% by 2030 [3]. Effective newborn resuscitation is clearly an important component in helping to ameliorate the odds of neonatal brain injury and improve survival. In order to improve standards around advanced newborn resuscitation, we must first ascertain the current timings of key events. Our aim was therefore to describe the real-life timings as to when certain key resuscitation events were achieved in a historic series of full newborn resuscitations.

## Methods

We conducted a retrospective case note review of babies inborn at our large UK university hospital which has approximately 6000 deliveries per year and provides tertiary-level neonatal intensive care. We reviewed cases of babies who had a 1 min Apgar score of 0 and who had undergone full resuscitation after birth. We defined a priori that a baby who received “full resuscitation” was one who needed all of the following: positive pressure ventilation (via face mask and/or endotracheal tube), cardiac compressions, and at least attempted central venous access for emergency drug administration. We identified cases by searching neonatal and maternity databases (BadgerNet Neonatal, Clevermed Ltd., UK; Euroking Maternity Software Solutions Ltd., UK) for the 10-year period January 2006 to December 2015. We excluded babies born at < 26 weeks’ gestation, those born outside of our centre, births following medical termination of pregnancy, and expected stillbirths. The neonatal resuscitation team comprised a minimum of three personnel: one neonatal nurse, one junior paediatric/neonatal trainee (1–3 years’ experience), and one senior paediatrician/advanced nurse practitioner (at least 4 years’ experience). All medical and nursing personnel involved in neonatal resuscitations were newborn life support (NLS) trained and UK Resuscitation Council certified, and undergo a refresher course and recertification every 4 years.

Using a dedicated pro forma, we interrogated the contemporaneous neonatal and maternal records for documented timings of key resuscitation events and outcomes.

## Results

Of 91 babies identified with an Apgar score of 0 at 1 min during the study period, 64 were excluded (42 did not meet the a priori definition of full resuscitation, 19 were born < 26 weeks’ gestation, and 3 were outborn). For the 27 babies included, the median birth gestation and birth weight were 36.1 weeks (range 27.2–41.2 weeks) and 2774 g (range 980–4778 g); 14/27 (52%) were born at ≥ 37 weeks’ gestation, 9/27 (33%) were born at 32–36 weeks’ gestation, and 4/27 (15%) were born at < 32 weeks’ gestation. Sixteen (59%) were male; delivery was by Caesarean section *n* = 16, normal vaginal *n* = 7, and instrumental *n* = 4. Arterial cord pH was median 6.95 (range 6.60–7.30) and base excess was median − 10.75 (range − 3.50 to − 29.0) mEq/L. Median Apgar score at 5 min was 0 (range 0–5) and at 10 min was 1 (range 0–6).

**Fig. 1:**
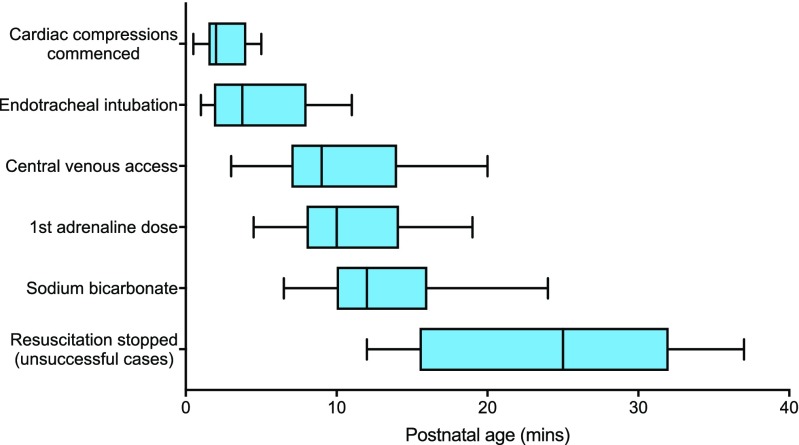
Timeline of key neonatal resuscitation events: box and whisker plot (medians, interquartile ranges, full ranges)

Table 1 presents the documented timings of key resuscitation events for the 27 included babies, and Fig. 1 represents the chronological sequence. For 8/27 (30%) resuscitations, the neonatal team was already in attendance at the time of delivery, whereas in 13/27 (48%) cases, the team was summoned after delivery and arrived at median 2.0 min (range 1.0–5.0 min); the timing of the team’s attendance was undocumented in 6/27 (22%) cases. There were no statistically significant differences in times by which the key events of umbilical venous catheter (UVC) insertion, intubation, and first adrenaline dose were achieved in resuscitations where the neonatal team was already present compared with those where the team needed to be summoned (data not shown). Of 25 babies who received intravenous adrenaline, only 10 (40%) received their first dose prior to 10 min of age. In four cases (15%), umbilical venous catheterisation proved difficult and adrenaline was first given via an alternative route: peripheral vein (*n* = 1), intra-osseous (n = 1) or endotracheally (*n* = 2). In two cases, adrenaline was not given as the clinical condition improved.

**Table 1:** Timing of key resuscitation events in 27 neonates who required full resuscitation at birth

Key event	Median postnatal age [95% CI], minutes	Event achieved but timing undocumented, *n* (%)
Neonatal team arrived (*n* = 27)	1.5 [0.0–3.0], (*n* = 21)	6/27 (22%)
Chest movement achieved (*n* = 26)	1.0 [1.0–2.0], (*n* = 15)	11/26 (42%)
Cardiac compressions commenced (*n* = 27)	2.0 [1.7–3.8], (*n* = 15)	12/27 (44%)
Endotracheal intubation achieved (*n* = 26)	3.8 [2.0–7.5], (*n* = 25)	1/26 (4%)
Central venous access achieved (*n* = 23)	9.0 [7.0–14.0], (*n* = 21)	2/23 (7%)
First dose intravenous adrenaline (*n* = 25)	10.0 [8.0–14.0], (*n* = 22)	3/25 (12%)
Second dose intravenous adrenaline (*n* = 19)	11.0 [10.0–19.0], (*n* = 15)	4/19 (21%)
First fluid bolus (*n* = 17)	13.0 [10.0–15.0], (*n* = 14)	3/17 (18%)
First dose intravenous sodium bicarbonate (*n* = 17)	12.0 [10.0–15.0], (*n* = 13)	4/17 (24%)
Time to first detectable heart rate (*n* = 19)	8.3 [5.0–13.9], (*n* = 15)	4/19 (21%)
Arrival of consultant (*n* = 20)	8.0 [5.0–14.0], (*n* = 17)	3/20 (15%)
First gasp (*n* = 20)	9.0 [1.0–14.0], (*n* = 11)	9/20 (45%)
Cardiac compressions ceased (*n* = 27)	16.3 [11.0–25.4], (*n* = 12)	15/27 (56%)
Ongoing resuscitation measures ceased (*n* = 9)	25.0 [15.8–26.5], (*n* = 9)	0/9 (0%)

9/27 (33%) babies showed no signs of life throughout and died despite all resuscitation attempts, 8 (30%) survived to neonatal unit admission but subsequently died before discharge, and 10 (37%) survived to discharge. Of these, 1 developed cerebral palsy and died in later infancy, while 9 survive long term and are presently aged 4–11 years (*n* = 4 had normal neurodevelopment at age 2 years; *n* = 4 have a poor outcome including cerebral palsy/global developmental delay/epilepsy or isolated severe bilateral sensorineural hearing loss; *n* = 1 is lost to follow-up).

## Discussion

Thorough and accurate documentation of key events during neonatal resuscitation is vital. The UK Resuscitation Council’s Newborn Life Support guidance advises documenting “what you did, when you did it, and the timing and details of any response from the baby” though steers clear of making recommendations for benchmark times by which key interventions should be achieved [8]. We believe our study is the first to focus on timings of achievement of a number of key resuscitation events in a historical series of real-life prolonged resuscitations. We found that timings of crucial resuscitation milestones were often unrecorded in the medical records, whereas in contrast the time of withdrawing resuscitation was uniformly well documented for all babies who died in the delivery room. Documented timings for commencement and ceasing of cardiac compressions and timings of first gasp and heart rate were especially poorly recorded, being absent in > 40% of cases. Such timings are important not only to help the perinatal teams and parents better understand the chronology of delivery room events, but will help make the medical record more robust if subject to future medicolegal scrutiny. We suggest that a standardised resuscitation proforma be developed that could be widely adopted and act as an aide memoire for assisting resuscitating practitioners with the contemporaneous recording of key events.

Currently, the International Liaison Committee on Resuscitation suggests that in “infants with an Apgar score of 0 after 10 minutes of resuscitation, if the heart rate remains undetectable, it may be reasonable to stop assisted ventilation.” [2] Only 40% of babies in our series received their first dose of adrenaline within the first 10 min after birth and the median time to withdrawal of resuscitation in non-survivors was 25.0 min (Fig. 1). That asystolic babies were regularly being resuscitated beyond 10 min, notwithstanding current advice, was probably not unreasonable considering that intravenous drug administration was frequently delayed. Our findings are comparable with those reported by another centre where 13 infants asystolic within the first 10 postnatal minutes had a median time of UVC insertion and adrenaline administration of 12 min [9]. Recent studies have suggested that resuscitation might be continued beyond 10 min, even if no heart rate has been detected by then, as outcomes may not be as universally poor as previously thought [9, 12].

The siting of a UVC in the emergency situation can be challenging. While one may be inserted in 105 s in a mannequin during a simulated resuscitation [7], we have shown that the real-life timings occurred much later (median 540 s). Where a UVC is proving difficult, intra-osseous (IO) access may be a useful option. IO safety has been demonstrated in term and preterm infants [4], and it is now recommended as a second-line form of access [8]. While IO insertion may be timely in comparison to UVC in the artificial setting [1, 7], there are no randomised-controlled trials of its efficacy against or alongside UVC insertion in neonates.

A limitation of our study is that it was retrospective and relied on timings of key resuscitation events as recorded during the high-stress setting of a delivery room resuscitation. While several cases reviewed had paper towels filed in the front of their case notes with obviously contemporaneous scribbled timings of events on them, in most cases it was unclear whether the timings had been recorded “live” during the resuscitation by an independent scribe, or whether they had been written retrospectively on reflection by the practitioner soon after the event. Resuscitating practitioners may underestimate the passage of time in neonatal resuscitation [11], which could lead to true timings being even later than documented. A future prospective study which aims to log the key resuscitation milestones contemporaneously and rigorously would be of interest, not least for comparison purposes with our retrospective data hereby presented.

## Conclusions

This study presents timings of key resuscitation milestones in a historic series of full newborn resuscitations that were conducted in the era of routine newborn life support training. Timings of important resuscitation events were often lacking in the contemporaneous medical documentation. The wide range of timings we present from real-life cases may prove useful to clinicians involved in medical negligence claims, and may provide a baseline for quality improvements in resuscitation training and medical documentation.

## Abbreviations

IO: Intra-osseous
NHS: National Health Service
UK: United Kingdom
UVC: Umbilical venous catheter
